# Gut microbiota-derived metabolites tune host homeostasis fate

**DOI:** 10.1007/s00281-024-01012-x

**Published:** 2024-07-11

**Authors:** Seungil Kim, Sang-Uk Seo, Mi-Na Kweon

**Affiliations:** 1 Mucosal Immunology Laboratory, Department of Convergence Medicine, University of Ulsan College of Medicine / Asan Medical Center, Seoul, Republic of Korea; 2 Digestive Diseases Research Center, University of Ulsan College of Medicine, Seoul, Republic of Korea; 3 Department of Microbiology, College of Medicine, The Catholic University of Korea, Seoul, Republic of Korea

**Keywords:** Gut microbiota, Metabolites, Mucosal immunity, Gut barriers

## Abstract

The gut microbiota, housing trillions of microorganisms within the gastrointestinal tract, has emerged as a critical regulator of host health and homeostasis. Through complex metabolic interactions, these microorganisms produce a diverse range of metabolites that substantially impact various physiological processes within the host. This review aims to delve into the intricate relationships of gut microbiota-derived metabolites and their influence on the host homeostasis. We will explore how these metabolites affect crucial aspects of host physiology, including metabolism, mucosal integrity, and communication among gut tissues. Moreover, we will spotlight the potential therapeutic applications of targeting these metabolites to restore and sustain host equilibrium. Understanding the intricate interplay between gut microbiota and their metabolites is crucial for developing innovative strategies to promote wellbeing and improve outcomes of chronic diseases.

## Gut microbiota–host immune interaction with metabolites

The human body hosts a wide array of commensal microorganisms, including bacteria, fungi, viruses, archaea, and protozoa [1–3]. Among this consortium, bacteria take precedence over other eukaryotic symbionts and have been extensively studied for their interactions with the host. The dominant bacterial phyla, Bacteroidetes and Firmicutes, alongside less abundant phyla such as Proteobacteria, Actinobacteria, and Verrucomicrobia, collectively constitute over 90% of microbiota in the gut. [4]. Initially recognized for their role in extracting energy from food, the metabolic contributions of gut microbiota have expanded to include fat storage and the regulation of glucose metabolism [5, 6]. Over the past two decades, advances in sequencing technologies have further revealed the association of gut microbiota with host homeostasis [7, 8]. The homeostatic gut microbiota fortifies the epithelial barrier against microbial invasion and promotes tolerance to innocuous luminal components, including the microbes themselves [9, 10]. However, disruptions in the gut microbiota consortium through broad-range antibiotic treatments render the host more vulnerable to inflammation [11, 12]. Research on germ-free mice has shown that the absence of gut microbiota impairs gut barrier function and renders animals susceptible to microbial invasions [13, 14]. As the essential role of gut microbiota in host immune homeostasis has been realized, the importance of understanding the mechanisms by which the host communicates with the gut microbiota has become pertinent. Recent advancements in multi-omics techniques have provided examples of host–microbiota interaction via diverse microbial products, including metabolites. Gut microbiota-derived metabolites, predominantly short-chain fatty acids (SCFAs), intermediate bile acid (BA) metabolites, amino acid-derived metabolites, and membrane-associated lipids, possess characteristics enabling communication with the immune system of their host (Table 1). These metabolites exert their biological activity through specific recognition by cognate receptors, primarily mediated by members of G-protein-coupled receptors (GPRs) [42]. GPRs exhibit distinct expression across various cell types, and the response to the same metabolite can vary based on specific cell roles [43]. Consequently, the response to gut microbiota-derived metabolites shows vast combinatorial diversity, posing substantial challenges in understanding their effects. Metabolites exhibit both paracrine and endocrine effects, enabling them to influence both local and systemic levels [44]. Systemically, metabolites can traverse the intestinal epithelium through active or passive transportation and directly impact organ function or contribute to diseases in distant tissues. In the gut–liver axis, BA processed by the gut microbiota can be transported back to the liver via the portal vein or disseminated to other body parts [45]. Gut microbiota-derived metabolites can also be carried to various tissues directly through the bloodstream, thereby influencing organ function and overall host physiology. Moreover, microbiota can generate metabolites that influence other microbiota members or pathogens, thereby modifying the overall composition or behavior of certain members. These changes can ultimately affect the host at the local and systemic level. This comprehensive review integrates up-to-date knowledge on the pivotal role of gut-microbiota-derived metabolites, emphasizing the need for further investigations to elucidate the complex mechanisms underlying their interaction with the host immune system.

**Table 1:** Different classes of microbial metabolites, receptors, and functions

Microbial metabolites	Receptor	Functions	References
Short-chain fatty acids			
Acetate	GPR41, GPR43	Treg homeostasis, IECs regeneration and differentiation ↑	[15, 16]
Propionate	GPR41, GPR43	γδ T cells ↓, IECs regeneration and differentiation ↑	[16, 17]
Butyrate	GPR41, GPR43, GPR109A	Treg ↑, IL-10 producing regulatory B cells ↑, Memory potential of CD8^+^ T cells ↑, Expression of tight junctional protein ↑, Myeloid homeostasis (Modulation of neutrophil and monocyte population), Alleviation of pancreatitis by enhancing gut integrity	[18–23]
SCFAs intermediates			
Lactate	GPR81	IECs regeneration and differentiation ↑, Support of hematopoiesis and erythropoiesis, Promote of circulating pathogen by Kupffer cells	[24–26]
Succinate	GPR91	Ileal inflammation ↓, Type2 immune response	[27, 28]
Lipid			
Membrane lipids	TLR1/2, TLR4	Inducing inflammatory cytokine as TLR2 agonist, Treg homeostasis, Anti-stomatitis virus and influenza virus	[29–32]
Saturated fatty acids	GPR40/120	Production of IFN-I from pDC and monocyte ↑	[33]
Conjugated linoleic acids	GPR40/120	Modulation of intraepithelial lymphocyte population Inflammation and allergy ↓	[34, 35]
Amino acid metabolite			
Tryptophan derivatives	Ahr	Production of IL-10 producing regulatory B cells, Gut barrier function, Ahr dependent IL-22 production in the gut mucosa	[18, 36, 37]
Bile acids			
IsoDCA	FXR	Treg homeostasis	[38]
IsoalloLCA	FXR	Treg homeostasis	[39]
3-oxoLCA	RORγt	Differentiation of Th17 ↓	[39]
LCA	TGR5	IECs regeneration and differentiation ↑	[40]
CDCA	FXR	Wnt2b production in cancer-associated fibroblast like colonic mesenchymal stem cell by BA-FXR axis	[41]

## Gut microbiota-derived metabolites

The diversity of gut microbiota-derived metabolites is vast, and their synthesis is intricately interconnected. Specific metabolites, such as SCFAs, lactate, succinate, amino acid-derived compounds, and BA, are pivotal to preserving homeostasis, despite belonging to distinct metabolic classes. In this review, we elucidate the primary types of gut microbiota-derived metabolites, focusing on those of particular relevance to host homeostasis.

### Short-chain fatty acids

SCFAs, also known as volatile fatty acids, are a category of fatty acid species containing fewer than six carbons that represent the most extensively studied metabolites in host–microbiota interactions. The biosynthetic pathways of SCFAs are closely integrated and share gut microbiota-derived monosaccharides as instigators. Each SCFA can be generated by multiple synthetic pathways provided by distinct microbiota sets. The biosynthetic pathway of SCFAs has been comprehensively reviewed previously [46]. While gut microbiota-derived SCFAs with one or five carbons, such as formate and valerate, are rarely recognized for their role in health and disease [47, 48], SCFAs produced by the microbiota, such as acetate (a two-carbon SCFA), propionate (a three-carbon SCFA), and butyrate (a four-carbon SCFA), are recognized for their involvement in various immunological and pathophysiological functions. These gut microbiota-derived SCFAs are absorbed into the bloodstream and have widespread effects on various cell types [49]. These effects occur through the interaction of SCFAs with specific receptors located on intestinal epithelial and immune cells. SCFAs are primarily produced in the colon due to the fermentation of indigestible dietary fiber, mainly by two anaerobic bacterial phyla: Bacteroidetes and Firmicutes [50]. Bacteroidetes, such as *Bacteroides* spp. play a significant role in the production of acetate and propionate [51]. Meanwhile, Firmicutes, exemplified by *Faecalibacterium prausnitzii*, are primarily responsible for generating butyrate [52].

### Lactate and succinate

Central carbon metabolism not only produces SCFAs as primary end-products, but also plays an important role in the production of crucial intermediate metabolites, including lactate and succinate. Lactate is generated in the gut through various pathways, including the fermentation of dietary fibers by gut microbiota and lactate production by epithelial cells [53, 54]. The gut microbiota can produce lactate through the fermentation of diverse substrates, such as glucose, fructose, and complex carbohydrates [55, 56]. Representative lactic acid-producing bacteria (LAB) include probiotics such as *Lactobacillus*, *Bifidobacterium*, *Lactococcus*, *Leuconostoc*, and *Weissella* [57]. Two types of LAB exist depending on the fermentation method. Homofermentative bacteria exclusively generate lactic acid as their main byproduct during glucose fermentation. In contrast, heterofermentative bacteria synthesize a substantial quantity of other metabolites, such as ethanol, acetic acid, propionic acid, acetaldehyde, or diacetyl, alongside lactate [58]. Lactate also significantly impacts the composition and diversity of the gut microbiota as it serves as a substrate for microbial growth and metabolism in the gut microbiota. Succinate functions as an intermediate metabolite in the production of propionate and is found in large quantities in intestinal luminal contents. Its levels significantly fluctuate under dysbiotic conditions, such as antibiotic treatment or inflammation, indicating a close association between succinate metabolism and the gut microbiota [59]. Although little is known about the microbial source of succinate, studies suggest that *Prevotella* is associated with its production in the human intestine [60]. Another study revealed that monocolonization of germ-free mice with *Bacteroides acidifaciens* increased intestinal succinate concentration compared with colonization with other gut microbiota, such as the *Clostridia* consortium [61].

### Bacterial membrane and dietary lipids

In addition to well-characterized smaller lipids, such as SCFAs and their intermediates, a significant proportion of larger lipid metabolites is produced by the gut microbiota. Some of these lipids are synthesized de novo and presented in the microbial membrane to influence host immunity. Most bacterial membrane lipids comprise fatty acid chains, also known as acyl chains, attached to distinct backbones. These acyl chains differ in length and saturation, giving rise to a variety of lipid species. The most recognized classes of membrane-bound lipids found in bacterial membranes include saccharolipids, phospholipids, sphingolipids, sulfonolipids, and cardiolipins [62–66]. The gut microbiota also plays a significant role in transforming, purifying, and digesting dietary fats, and the resulting products can impact both local and systemic homeostasis [67]. For instance, the Bacteroidetes phylum transforms dietary sphingolipid and cholesterol using their enzymes [68–70], while *Bifidobacterium* expresses enzymes that break down complex sphingolipids [69]. *Lactobacillus* and *Bifidobacterium* convert linoleic acid to conjugated linoleic acid (CLA) using their enzymes [71], and dietary polyunsaturated fatty acids (e.g., $\omega$-6 and -3 fatty acids) can be modified by gut microbiota-derived enzymes, causing changes in the gut microbiota composition [72–74]. *Clostridium butyricum* induces $\omega$-3 fatty acid production in the gut [75], while saturated fatty acids, such as oleic and palmitic acid, are produced by *Bacteroides thetaiotaomicron*, *Lactobacillus johnsonii*, and *Lactobacillus paracasei*, inhibiting viruses and fungi [33, 76].

### Amino acids-derived metabolites

Due to the substantial presence of gut microbiota in the intestine, their integral metabolism of amino acids results in the production of a wide range of related metabolites, including ammonia, amines, nitrogen compounds, phenols, and precursors to SCFAs [77]. Byproducts originating from aromatic amino acids such as tryptophan, tyrosine, and phenylalanine give rise to a diverse array of phenolic and indolic compounds through a series of alterations [78]. Tryptophan, an aromatic amino acid, undergoes metabolism by gut microbiota, leading to the formation of its derivatives, such as indole, indolepropionic acid (IPA), indoleacrylic acid (IAA), 3-methylindole (Skatole), indolealdehyde (IAld), indoleethanol (IE), indolelactic acid (ILA), and tryptamine [79]. Whereas bacteria belonging to Actinobacteria, Firmicutes, Bacteroidetes, and Proteobacteria produce a variety of tryptophan metabolites with different genera, yielding distinct types of these metabolites [79, 80]; for example, *Clostridium sporogenes* and *Clostridium botulinum* produce IPA [81, 82]. Meanwhile, broader gram-positive and gram-negative gut microbiota, such as *Enterococcus faecalis* and *Escherichia coli*, produce indole, in addition to Firmicutes [83, 84]. Additionally, l-carnitine, an amino acid abundant in red meat, is converted to trimethylamine (TMA) by the gut microbiota [85]. TMA is delivered to the liver, where it is then converted to trimethylamine N-oxide (TMAO), accelerating atherosclerosis in mouse models.

### Bile acids

BAs, synthesized in the liver, are released into the small intestine to facilitate the digestion and absorption of dietary fats. The liver produces primary BAs by converting cholesterol through the enzyme cholesterol 7α-hydroxylase (CYP7a1) [86]. The two main BAs, chenodeoxycholic acid (CDCA) and cholic acid (CA), conjugate with glycine or taurine to form glycol-conjugated or tauro-conjugated BAs. Conjugated primary BAs are vital in the digestion and absorption of lipids and vitamins [87]. Approximately 95% of primary BAs are actively reabsorbed from the ileum and undergo recycling within the liver, a process known as enterohepatic circulation [88]. Primary conjugated BAs can also undergo further modification by the gut microbiota. Initially deconjugated, they are subsequently transformed into various secondary BAs, such as deoxycholic acid (DCA), lithocholic acid (LCA), and ursodeoxycholic acid (UDCA). These secondary BAs are then passively reabsorbed into the circulating BA pool or eliminated through feces. Meanwhile, a portion of both primary and secondary BAs enters systemic circulation, exerting biological functions in distal parts of the body [89]. To generate secondary BAs, gut microbiota must express bile salt hydrolases (BSHs), necessary for deconjugation, and multiple transforming enzymes [90, 91]. Bacteria belonging to Firmicutes, Proteobacteria, Fusobacterium, Bacteroidetes, and Actinobacteria express these enzymes; however, the types of secondary BAs they produce vary according to the diversity of BSH-encoding genes and specific dehydrogenase they express [91, 92].

## Receptors for gut microbiota-derived metabolites

### G protein-coupled receptors

GPRs constitute a class of cell membrane receptors that play a fundamental role in cell signaling [93]. They are involved in various physiological processes, including the regulation of neurotransmission, hormonal signaling, immune responses, and the perception of taste and odor. Abundantly present throughout the body, GPRs are also found in the gastrointestinal tract. Endeavors to identify the functions of orphan GPRs have revealed interactions between several selective GPRs with various metabolites [42]. These metabolites, acting as signaling molecules, include substances such as SCFAs and BAs, some of which are derived from the gut microbiota, and play a role in triggering classical signal transduction processes. GPRs specific to gut microbiota-derived metabolites include GPR40, GPR41, GPR43, GPR81, GPR91, GPR109A, and GPR120, which are classified into four families according to their alpha subunit (Gi, Gs, G12/13, and Gq) [94–96]. Most GPRs recognizing metabolites derived from the gut microbiota have Gi or Gq-coupled receptors. For instance, GPR41, GPR81, and GPR109A comprise Gαi receptors, while GPR40, GPR43, and GPR91 have Gαi/Gαq, and GPR120 has Gq. The Gi family modulates adenylyl cyclase, and Gq stimulates phospholipase C [42]. Specific GPR ligands exhibit bias by selectively activating particular pathways downstream of a GPR. These biased ligands target only a subset of these pathways, leading to a more focused and specific cellular response [97]. For instance, GPR40, proposed as a potential target for diabetes treatment, functions as a Gq-coupled receptor responsive to long-chain fatty acids (LCFA). When triggered by specific agonists, GPR40 can initiate signaling not only through Gq and IP3 but also via Gs and cAMP, setting it apart from natural LCFA ligands [98].

### Pattern recognition receptors

Membrane lipids distinguish themselves among microbial metabolites by virtue of their exclusive presence in microbes, rendering them potent pathogen-associated molecular patterns (PAMPs). They serve as distinctive microbial signatures that can be readily recognized by the host's pattern recognition receptors (PRRs), such as Toll-like receptors (TLRs). A notable example is lipopolysaccharide (LPS), a prominent member of the saccharolipid class produced by a broad spectrum of gram-negative bacteria [99]. The recognition of LPS by TLR4 initiates a cascade of reactions that can result in overt inflammation. However, this inflammation can, at times, be beneficial in preserving homeostasis because weak TLR4 stimuli can induce tolerogenic immune cell activation, thereby restoring disrupted intestinal immune balance [100]. Similarly, phospholipids produced by *A. muciniphila* can stimulate TLR1/2 to induce homeostatic immunity [29]. There is a possibility that the continuous interaction between coexisting gut bacteria and their inherent PAMPs has evolved towards maintaining immunity rather than over-activating it. Hence, further research into the immunoregulatory function of PRRs holds substantial promise for our understanding and the development of strategies to maintain immune balance in the future.

### Aryl hydrocarbon receptor

The host experiences a diverse array of effects when tryptophan-derived metabolites engage with the aryl hydrocarbon receptor (AhR), a nuclear receptor [101]. Tryptophan derivatives, such as indole propionic acid, indole-3-aldehyde, indole lactic acid, indole pyruvate, and indole acetic acid, act as microbiota-derived AhR ligands [79]. In the absence of a ligand, AhR exists in the cytosol as a protein complex comprising HSP90 (90 KDa heat shock protein), AhR-interacting protein (AIP), cochaperone p23, and protein kinase c-Src [102]. After ligand binding, a conformational change induced in AhR leads to the dissociation of the complex and nuclear translocation. In the nucleus, a series of downstream gene expressions occur. The activation of the AhR signaling pathway holds promise in alleviating inflammation-related disorders, such as ulcerative colitis [103], and regulation of gut motility by modulating enteric neurons, potentially bolstering mucosal barrier function [36, 37, 104, 105].

### Farnesoid X receptor and Takeda G protein-coupled receptor 5

The Farnesoid X Receptor (FXR) is a nuclear receptor protein that is critical in the regulation of various metabolic processes throughout the body [106]. Primarily located in the liver, intestine, and other tissues involved in BA metabolism and lipid homeostasis, FXR acts as a transcription factor, controlling the expression of specific genes by binding to their regulatory regions [107]. Its primary function is to regulate the synthesis of BAs, which, in turn, reduces the expression of CYP7a1 by triggering hepatic FXR [108]. Similarly, in the small intestine, activated FXR hinders BA synthesis by releasing fibroblast growth factor (FGF) 15/19 [109]. FGF15/19 then moves to the liver, where it activates FGF receptor 4 on hepatocytes and causes suppression of CYP7a1 expression. FXR also has a broader role in regulating glucose and lipid metabolism, including influencing the expression of lipid and glucose homeostasis-related genes in the liver and adipose tissue [110]. Takeda G protein-coupled receptor 5 (TGR5) is found on cell surfaces and activated by BAs [111]. It is present in different body tissues such as the gallbladder, intestine, and brown adipose tissue. When BAs bind to TGR5, they initiate internal signaling pathways that control several bodily functions, including energy processing, glucose level maintenance, and immune system responses [112–114]. BAs act as signaling molecules by activating both TGR5 and FXR receptors, regulating various metabolic processes, and maintaining BA homeostasis. The interaction between these receptors and BAs is crucial for preserving metabolic balance and regulating diverse physiological functions throughout the body.

## Impact of gut microbiota-derived metabolites on host homeostasis

### Modulation of mucosal immune cell functions

Microbiota-produced metabolites act as signaling molecules, engaging with host cells, especially immune cells, at mucosal surfaces [115]. These metabolites directly impact immune cell behavior, influencing factors such as cell activation, differentiation, migration, and cytokine production. By interacting with receptors on immune cells, microbiota-derived metabolites enhance or suppress immune responses depending on the specific metabolite and context. This finely tuned modulation is essential for maintaining balance in the immune system. SCFAs wield significant influence on immune cells in the gut mucosa, which affects their activation and regulation, particularly the differentiation and activation of regulatory T (Treg) cells [15, 116]. Dietary fiber and bacterial SCFAs also heighten the activity of tolerogenic dendritic cells and expression of retinaldehyde dehydrogenase, leading to increased Treg differentiation [117, 118]. Propionates suppress IL-17 and IL-22-producing intestinal γδ T cells through a histone deacetylase-dependent mechanism [17]. Butyrate, derived from gut microbiota, alleviates arthritis by boosting the production of IL-10-producing regulatory B cells. It achieves this by increasing the concentration of the tryptophan-derived metabolite 5-hydroxyindole-3-acetic acid, which activates the AhR [18]. Butyrate also enhances the memory potential of CD8^+^ T cells [19]. SCFAs also modulate dendritic cell function by promoting their maturation, enhancing antigen presentation, and influencing cytokine production [119, 120]. Microbiota-produced succinates contribute to the expansion of tuft cells and the suppression of ileal gut inflammation [27]. These microbial succinates also activate tuft cells and initiate a type 2 innate immune response in the small intestine [28]. Microbiota-derived BAs influence colonic Treg homeostasis [121, 122]. For instance, BA isoDCA restricts the activity of FXR in dendritic cells, conferring an anti-inflammatory phenotype and enhancing the induction of Foxp3 in Treg cells [38]. 3-OxoLCA impedes the development of Th17 cells by interacting directly with the crucial transcription factor RORγt, while isoalloLCA encourages the formation of Treg cells [39]. Gut microbiota-derived lipid metabolites also have immunomodulatory effects. Metabolites such as 9, 10-dihydroxy-12Z-octadecenoic acid, and all-trans retinoic acid from specific-pathogen-free mice induce Treg activity [30]. Moreover, conjugated linoleic acid (CLA) derived from gut microbiota promotes the induction of a CD4^+^ intraepithelial lymphocyte population expressing CD8αα in the small intestine [34]. *Bacteroides fragilis* sphingolipids regulate colonic iNKT cell homeostasis and shield against colitis challenges [123]. Additionally, cell surface polysaccharides derived from *Bifidobacterium bifidum* stimulate Treg cells, effectively suppressing experimental colitis [31]. Overall, metabolites derived from the gut microbiota collectively play a crucial role in modulating the function and equilibrium of mucosal immune cells.

### Modulation of intestinal barrier function

**Fig. 1:**
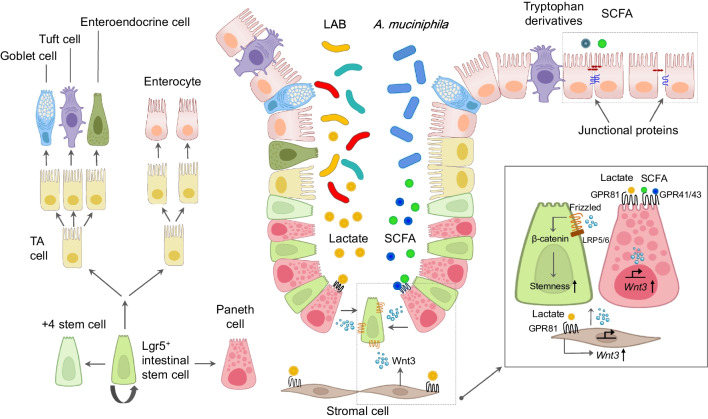
The process of maintaining the balance in the renewal and differentiation of intestinal epithelial cells. Lgr5^+^ stem cells reside adjacent to Paneth cells at the base of the crypt. These Lgr5^+^ stem cells constantly generate rapidly proliferating TA cells, filling the crypt. Subsequently, TA cells undergo differentiation into various functional cell types present within the villi, including enterocytes, tuft cells, goblet cells, and enteroendocrine cells. Lactic acid-producing bacteria, such as *Bifidobacterium* and *Lactobacillus*, contribute to the renewal of intestinal epithelial cells. The lactate produced by these symbiotic organisms is recognized by the GPR81 on Paneth and stromal cells, facilitating regeneration through the Wnt3/β-catenin pathway. The mucin-degrading bacterium *A. muciniphila* promotes intestinal stem cell-mediated epithelial regeneration using metabolites such as SCFA within the Wnt3/β-catenin pathway. Gut microbiota-derived tryptophan derivatives, along with SCFA, stimulate the expression of junctional proteins (such as occludin, claudins, and zonula occludens), thereby enhancing the gut barrier functions

The intestinal barrier comprises three protective layers that prevent the entry of bacteria from the gut lumen [124]. These layers include the luminal mucus layer, a continuous sheet of gut epithelial cells, and an inner layer responsible for forming the mucosal immune system. Metabolites derived from the microbiota help maintain the integrity of the mucosal barrier [125]. They aid in enhancing mucus production and tightening epithelial cell junctions, preventing the entry of harmful substances. Some metabolites also support tissue repair and regeneration, crucial for the quick recovery of mucosal surfaces after damage or inflammation [126]. By providing both physical and immunological defense mechanisms, this barrier is critical in defending against luminal microorganisms, viruses, food antigens, and environmental toxins. The gut epithelial barrier comprises a single layer of intestinal epithelial cells (IECs) that renew every 3–5 days. These cells originate from intestinal stem cells (ISCs), expressing leucine-rich repeat-containing G-protein-coupled receptor 5 (Lgr5) on their surface (Fig. 1). These ISCs give rise to ISC daughters or transit-amplifying (TA) cells [127]. TA cells then eventually undergo terminal differentiation and develop into absorptive or secretory cell lineages, including enterocyte, Paneth, goblet, enteroendocrine, and tuft cells. Our group has documented the interaction between microbiota-derived metabolites and the development of IECs. Lactates, produced by probiotics such as *Lactobacillus* and *Bifidobacterium* boost the proliferation of Lgr5^+^ ISCs through the Wnt/β-catenin pathway in Paneth cells and intestinal stromal cells [24] (Fig. 1). Moreover, lactates influence ISC-mediated epithelial development in a GPR81-dependent manner. *A. muciniphila* is recognized as a prominent symbiont and next-generation probiotic due to its health-promoting effects [128]. Interestingly, during the process of mucin degradation *A. muciniphila* also paradoxically enhances mucin production [16]. Moreover, it stimulates the regeneration of Lgr5^+^ ISCs and facilitates the differentiation of secretory lineage cells, including Paneth and goblet cells, within the gut. This process involves the utilization of metabolites such as acetate and propionate through GPR41/43 signaling. Additionally, BAs, specifically LCA, activate IEC regeneration via the BAs-TGR5 axis [40]. IECs establish connections through junctional proteins, including tight junction proteins, adherens junction proteins, gap junction proteins, and desmosomes [129]. The relationship between microbiota-derived metabolites and tight junctions is complex and bidirectional. These metabolites impact the expression and function of proteins associated with tight junctions, and assist in fortifying the assembly and integrity of these junctions, contributing to a more robust gut barrier. Various gut microbiota-derived metabolites, such as butyrate, quercetin, indole 3-propionic acid, tryptamine, and L-homoserine, can trigger an increase in the production of these junctional proteins [20, 130, 131]. Conversely, imbalances in the microbiota and their metabolites can affect tight junction function. Dysbiosis has been associated with changes in tight junction integrity and increased intestinal permeability [132], allowing harmful substances to translocate into the bloodstream, leading to inflammation and the development of metabolic diseases. Research suggests that conditions such as leaky gut syndrome, disruption of gut barrier function, and endotoxemia originating from the gut microbiota may contribute to the development of obesity and type 2 diabetes [133, 134]. A Western-style diet has been shown to increase gut permeability and reduce the expression of tight junction proteins, such as zonula occludens-1 and occludin, in the intestinal epithelial cells of mice [135]. In obese mice induced either genetically or by a high-fat diet, disruption of the gut barrier increases permeability, resulting in the release of LPS into the portal blood circulation [136, 137]. Multiple studies indicate that LPS plays a crucial role in initiating obesity and developing type 2 diabetes, with elevated LPS levels observed in obese and diabetic patients, suggesting its role as a marker for decreased intestinal barrier function [138]. Furthermore, mice subjected to a high-fat diet demonstrated that dysfunction in the intestinal barrier leads to increased disorders related to glucose dysmetabolism and liver steatosis [139].

### Gut–bone marrow interaction

**Fig. 2:**
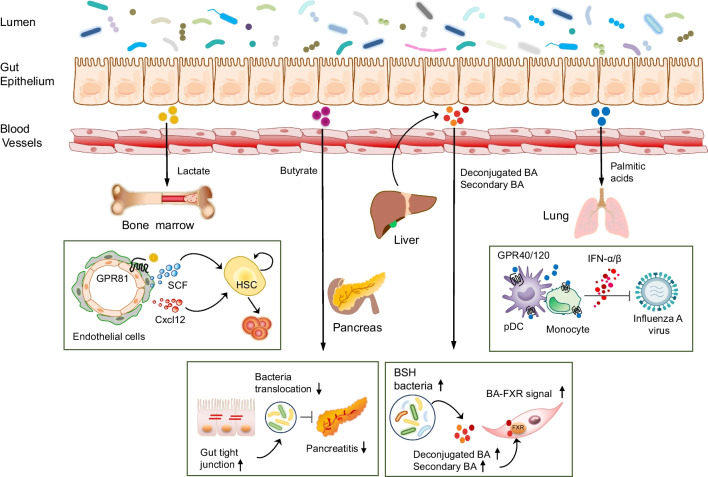
Cross-talk between gut microbiota-derived metabolites and various organs. Microbial byproducts, such as lactate, traverse through the bloodstream to reach the bone marrow, triggering stromal cells to release SCF and Cxcl2 influenced by GPR81 signaling. This interaction impacts bone marrow functions. Butyrate, generated by the gut microbiota, reinforces the gut barrier, fortifies the host against pathogen infiltration, and alleviates the effects of pancreatitis. Certain bacteria producing BSH elevate levels of deconjugated and secondary BAs within the colon. These BAs stimulate the production of Wnt2b in colonic mesenchymal stem cells through FXR activation, promoting increased cell growth in colonic crypts. Palmitic acid derived from *Lactobacillus* exhibits the ability to enhance the production of type-I IFN from pDC and monocytes. This increase in fatty acids contributes to the host's defense against influenza infection through the GPR40/120 mechanism

The bone marrow, located within bone cavities, contains pluripotent hematopoietic stem cells (HSCs) that differentiate into lymphoid progenitor or myeloid progenitor cells, which play a crucial role in the immune response, oxygen transport, and blood clotting. The bone marrow is therefore a central component of the circulatory and immune systems. The "gut–bone marrow axis" implies that there is a dynamic interplay between the gut and the bone marrow extending beyond their individual functions (Fig. 2). Recent research has revealed that substances produced by the gut microbiota, such as metabolites and signaling molecules, can impact the activity and function of bone marrow cells [25, 140]. Lactate produced by probiotics enters the bloodstream and reaches the bone marrow, where it stimulates the expression of stem cell factors necessary for the proliferation of HSCs in a GPR81-dependent manner [25]. These findings underscore a crucial link between microbiota-produced lactate and the expansion of HSCs, thereby providing continuous support for hematopoiesis and erythropoiesis. Moreover, increased myeloid cell differentiation from HSCs, prompted by microbiota-derived LPS stimulation, leads to higher and more sustained levels of IL-1α and IL-1β in the bone marrow [140]. Conversely, the bone marrow can reciprocally influence the gut by producing immune cells and factors that regulate gut inflammation [141, 142]. Bone marrow transplantation induces marked changes in microbiota composition and impacts the severity of intestinal graft-versus-host disease [141]. Transferring bone marrow from mice colonized with *Clostridium scindens* protects naive mice against colitis induced by *Entamoeba histolytica* [142]. Signals derived from commensals influence basophil hematopoiesis and susceptibility to allergic inflammation [143]. Peptidoglycan from the gut microbiota regulates the steady-state lifespan of neutrophils and monocytes, contributing to myeloid homeostasis [144]. Additionally, microbiota-derived compounds maintain steady-state granulopoiesis through MyD88 signaling [145]. Butyrates derived from the gut microbiota alter hematopoiesis by increasing patrolling Ly6c^−^ monocytes in the context of influenza virus infection [21]. The microbiota regulates bone marrow function via butyrates and controls HSC self-renewal in an iron-dependent manner [22].

### Gut–lung interaction

Recent findings have illuminated the intricate communication network between the gut microbiota and the respiratory system, often referred to as the gut–lung axis. This multifaceted framework encompasses the gut microbiota, immune responses, inflammatory signaling, and dietary influences to link the gut and the lungs [146]. The dynamic interplay between these distant anatomical sites underscores the potential influence of metabolites originating from the gut microbiota on immune responses in the lungs [147]. Gut signals from Proteobacteria direct the migration of group 2 innate lymphoid cells from the gut to the lung via the gut-lung axis [148]. During an infectious state, gut microbiota-derived metabolites are crucial in protecting the host. Palmitic acid, derived from *Lactobacillus paracasei*, increases the production of type-I IFN from plasmacytoid DC and monocyte [33], which helps protect the host against influenza infection through a mechanism relying on GPR40/120. Metabolites derived from *Clostridium orbiscindens* in the human gut, such as desaminotyrosine, protect against respiratory virus infection by activating type-I IFN in macrophage [149], while glycolipids associated with *Bacteroides fragilis* confer resistance to vesicular stomatitis virus and influenza virus by type-I IFN producing colonic dendritic cells [32]. Additionally, the presence of SCFAs produced through bacteria-induced inulin fermentation increases the numbers of patrolling monocytes and enhances the effectiveness of CD8^+^ T cells, thereby mitigating the lung damage associated with influenza virus infection [21]. Furthermore, SCFAs help defend against allergic inflammation in the lungs by promoting the development of dendritic cell precursors through hematopoiesis [150]. Supplementation of CLA produced by gut microbiota, such as *Bifidobacterium* and *Lactobacillus*, reduces inflammation in individuals with allergies by reducing TNF-α and IFN-γ production from peripheral blood mononuclear cells [35].

### Gut–liver interaction

The gut–liver interaction is a multifaceted and dynamic relationship characterized by intricate biochemical, immunological, and endocrine communication. The balance and synergy between the gut and the liver play a pivotal role in BA metabolism, digestion, immune regulation, hormonal signaling, and overall health [151]. The portal vein receives most of the blood from the small and large intestine, carrying nutrients absorbed from the digestive tract and metabolites derived from the microbiota [152]. For example, gut microbiota-derived D-lactate arriving at the liver via the portal vein promotes the killing of circulating pathogens by Kupffer cells [26]. BAs modulate the composition of gut microbiota by utilizing their detergent properties to disrupt bacterial membranes [153], and induce stress, including DNA damage and protein misfolding [154]. BAs also activate FXR in IEC, promoting the production of antimicrobial molecules and inhibiting bacteria overgrowth [155]. Tauro-cholic acid and β-tauro-muricholic acid accelerate postnatal microbiota maturation in newborn mice [156]. Dietary signals influence BA-mediated gut microbiota composition and homeostasis, with a high-fat diet shown to increase the levels of primary BAs (e.g., chenodeoxycholic acid) and bacteria-producing BSHs [41]. These bacteria upregulate secondary BAs, which induce the production of Wnt2b through mesenchymal stem cells in the colon in a BA–FXR-dependent manner. Furthermore, BSH-producing bacteria and retinol modulate BA production in the gut in an intestinal liver kinase B1-dependent manner [157].

### Gut–pancreas interaction

The pancreas is connected to the gastrointestinal tract through the pancreatic duct system, creating an inherent association with the gut microbiota. In its healthy state, the pancreas does not directly interact with the gut microbiota and is traditionally not believed to have its own microbiome. Nevertheless, gut bacteria can migrate into the pancreas and potentially affect the pancreatic environment, even in individuals with a healthy pancreas. Dysbiosis has been detected in pancreatic disorders and could contribute to the development of various pancreatic conditions such as acute and chronic pancreatitis, and pancreatic cancer [158, 159]. Gut microbiota can also be directly transported through the pancreatic ductal system [160], a process facilitated by dysbiosis through the disruption of mucosal immune function and increase in gut permeability [161]. A recent study also validates the concept of gut microbiota-produced butyrate enhancing the integrity of the gut barrier, protecting the host from pathogen invasion and alleviating pancreatitis [23]. Patients with pancreatitis who received probiotics such as *Lactobacillus plantarum* showed improved gut permeability and clinical outcomes compared with the control group [162]. Additionally, administrating *Lactobacillus plantarum* and oat fiber to these patients effectively reduced pancreatic sepsis [163]. In pancreatic cancer, the gut microbiota modulates the tumor microbiome landscape and influences clinical outcomes [164]. Gut microbiota therefore plays a crucial role in pancreatic health and disease.

## Therapeutic implications and future perspective

The therapeutic landscape is actively exploring the potential of gut microbiota-derived metabolites across various clinical domains. Probiotics, encompassing distinct bacterial strains, are currently used to modulate the gut microbiota and stimulate the production of beneficial metabolites. Concurrently, dietary interventions, prebiotics, and postbiotics are emerging as promising therapeutic strategies to target these metabolites for therapeutic purposes. As this field matures, we could expect to see: (1) personalized therapies: tailoring treatments to the unique gut microbiota composition of an individual to optimize the production of beneficial metabolites; (2) advanced metabolomics: ongoing advancements in metabolomics technologies will furnish a more comprehensive understanding of the diverse spectrum of metabolites originating from the gut microbiota; (3) microbiome engineering: pioneering approaches to engineering the gut microbiota, driving production of specific metabolites for therapeutic utility. The intricate interplay between gut microbiota-derived metabolites and host homeostasis represents a flourishing area brimming with therapeutic promise. Harnessing these metabolites stands to unlock novel interventions across a broad spectrum of health conditions. The future holds the promise of innovative therapies leveraging the extraordinary capabilities of the gut microbiota in shaping host health and patient outcomes.

## Conclusion

This comprehensive review aims to consolidate current knowledge regarding the role of gut microbiota-derived metabolites in shaping host homeostasis. By elucidating the mechanisms of action and the impact on various physiological processes, this paper provides a foundation for future research and therapeutic interventions targeting the gut microbiota and its metabolites. Understanding the intricate relationship between gut microbiota and host physiology is essential for advancing our knowledge of health and disease and has the potential to revolutionize medical interventions.

## Data Availability

Data sharing not applicable to this article as no datasets were generated or analyzed during the current study.
